# Web Apollo: a web-based genomic annotation editing platform

**DOI:** 10.1186/gb-2013-14-8-r93

**Published:** 2013-08-30

**Authors:** Eduardo Lee, Gregg A Helt, Justin T Reese, Monica C Munoz-Torres, Chris P Childers, Robert M Buels, Lincoln Stein, Ian H Holmes, Christine G Elsik, Suzanna E Lewis

**Affiliations:** 1 Genomics Division, Lawrence Berkeley National Laboratory, Berkeley, CA 94720, USA; 2 Division of Animal Sciences, University of Missouri, Columbia, MO 65211, USA; 3 Department of Bioengineering, University of California Berkeley, Berkeley, CA 94720, USA; 4 Ontario Institute of Cancer Research, MaRS Centre, South Tower, 101 College Street, Suite 800 Toronto, Ontario, Canada M5G 0A3; 5 Department of Molecular Genetics, University of Toronto, 172 St. George Street Toronto, Ontario, Canada M5R 0A3; 6 Division of Plant Sciences, University of Missouri, Columbia, MO 65211, USA

**Keywords:** GENOME, COLLABORATIVE, EDITOR

## Abstract

Web Apollo is the first instantaneous, collaborative genomic annotation editor available on the web. One of the natural consequences following from current advances in sequencing technology is that there are more and more researchers sequencing new genomes. These researchers require tools to describe the functional features of their newly sequenced genomes. With Web Apollo researchers can use any of the common browsers (for example, Chrome or Firefox) to jointly analyze and precisely describe the features of a genome in real time, whether they are in the same room or working from opposite sides of the world.

## Introduction

The multitude of genome browsers in genomics all grew out of the need to 'see' the full array of predictions and alignments, their relative positions and their component parts. Among these are a small number of more sophisticated genome 'editors' which allow users to go beyond passive viewing to interactively modifying and refining precise locations and structures of genome functional elements. The desktop version of Apollo [1], Artemis [2], and FMAP [3] are all examples of such tools. The genome sequencing and annotation paradigm typically involved a large, national genome center that undertook the raw sequencing in coordination with gene prediction pipelines and subsequent manual curation (for example, RefSeq [4], Ensembl [5], FlyBase [6], Wormbase [7], *Saccharomyces *Genome Database [8], The Arabidopsis Information Resource [9], and Mouse Genome Informatics [10]). The Model Organism Databases (MODs) often include staff members (that is, biocurators) who review and amend the gene structures. The Human and Vertebrate Analysis and Annotation (HAVANA) team at the Sanger Institute manually annotates the human [11], mouse [12], and zebrafish [13] genomes. The amended predictions are subsequently used either as training sets or as empirical standards whose alignments are used to improve prediction software's accuracy. For example, the HAVANA team uses their in-house genome editor (Otterlace [14]) to manually annotate, and then the improved annotations are fed back into the Ensembl [15] pipeline during subsequent quarterly runs [16].

Unfortunately, while this model of a central biocuration team is considered the gold standard for genome annotation, it scales poorly. Technical advances have made sequencing faster and cheaper, thereby democratizing genome-scale sequencing and allowing a rapidly growing number of researchers to launch sequencing projects ranging from population, to evolutionary, to phenotype, to disease, to classroom projects across a huge spectrum of organisms. And, while next generation sequencing technology provides annotators with significantly more information, this, perhaps paradoxically, actually increases the need for manual review because there are more biological data points to assess and integrate. Individual researchers and small research groups do not have access to a centralized biocuration team, but their need for hand curation is often greater than that of a large genome center due to their focused interest in a particular gene family, pathway or evolutionary relationship, and the generally lower quality of the genome assembly.

An ideal solution would conceptually be a 'genome wiki', where curators could collaboratively edit genome annotations online, much like the distributed curators of a wiki document [17]. Biological text corpora, successfully exposed to 'crowd-sourced' curation via the wiki-type model, include Wikipedia pages directly associated with human genes [18] along with pages for protein [19] and RNA domain families [20]. Other projects offer similar wiki-like editing features for text, including revision control [21]. However, while editable textboxes have been present in browsers since the earliest days of the web, a completely integrated genome editor that operates seamlessly in the web browser (and saves annotations to a persistent data store in a client-server model) has been lacking. The natural user interface for genome data is the genome browser, and a true 'genome wiki' should allow curators to edit annotations seamlessly from within the genome browser.

**Figure 1:**
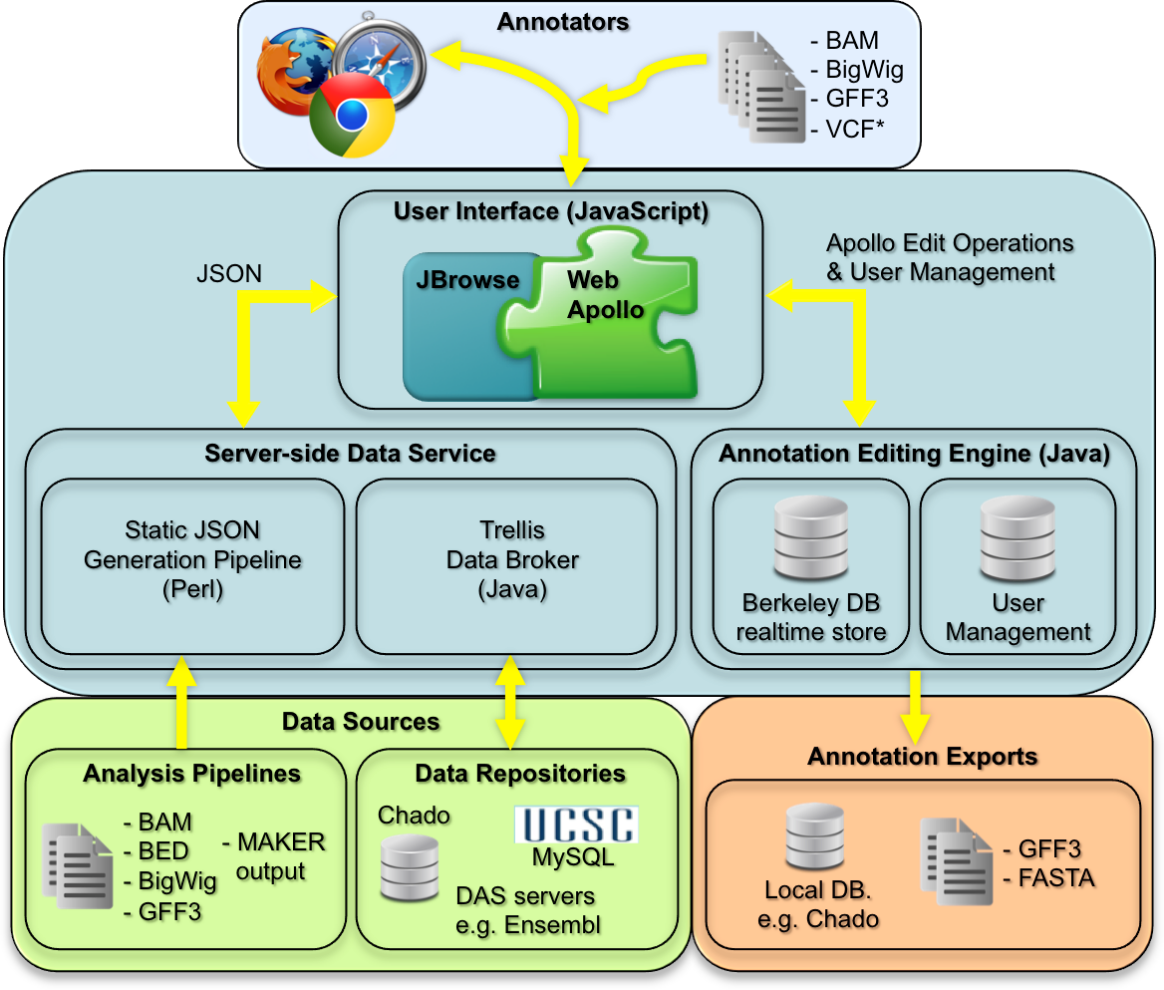
**Web Apollo architecture**. Web Apollo (components within the central turquoise box) acts as a mediating agent between users (top blue box) and external sources and sinks of data (lower green and peach boxes). Two user interface components operate on the client-side, within the browser environment. The JBrowse component visualizes various DNA features, and the Web Apollo component captures user manipulations. The Data Services module dynamically delivers genomic data and features to the user interface as JBrowse compatible JSON. Most of the primary genomic data is harvested and formatted in advance as part of the initial server setup. In addition, data from other sources may be dynamically provided using the Trellis framework or uploaded by the user from the browser. The Annotation Editing Engine and User Management components also sit on the server side. The first responds to users actions on the client by modifying the underlying data models appropriately, and second manages user accounts and login services. Annotations created by users can be exported as either GFF3 or FASTA file, or directly saved to a Chado database (plug-in adapters may be added to export genomic annotations to additional repositories). The arrows indicate where there are interactions between components, with the arrowhead indicating the direction of data flow.

For this reason, we built Web Apollo, a browser-based genome editor that supports geographically dispersed researchers whose work is coordinated through automatic synchronization. The overall platform is currently comprised of a visualization component - JBrowse [22], and an editing and user authentication component - Apollo. Just as the costs of sequencing have gone down our aim was to make manual annotation correspondingly cost-effective. With Web Apollo the task of manual curation is spread out among many hands and eyes, enabling the creation of virtual research networks of researchers linked by a common interest in a particular organism or population (Figure 1).

Our development team included investigators representing multiple genome research communities who carried out usability testing to evaluate the effectiveness of Web Apollo's interface and annotation management. We took this user-centered-design approach to ensure real world usability was built into the system from the ground up. They evaluated usability by revising annotations for honeybee (*Apis mellifera*) and, from the outset, for community annotation of insect genomes such as ants (*Cardiocondyla obscurior, Pogonomyrmex barbatus*, and *Wasmannia auropunctata*), leading to a better understanding of the biology of these insects and simultaneously evaluating the effectiveness of the software.

## Results

This section briefly explains Web Apollo's core operations for importing data, editing, and exporting protein-coding gene models. Additionally we describe additional features supporting the annotation of corrections to lower quality genome assemblies, import and visualization of transcriptome data, and real-time collaboration.

### Protein-coding gene annotation

To annotate a gene, curators commonly proceed by: (1) locating the region of interest; (2) inspecting all available gene predictions and biological evidence aligned to the region; (3) creating a gene model; (4) if necessary, modifying these gene models using the editing functions; (5) corroborating the accuracy of the annotation by comparing the resulting annotation with available homologs; and (6) ensuring that correct naming conventions and relevant comments have been added, utilizing available literature as needed.

*Importing genomic data*: Using server-side middleware, the system can load data tracks from a variety of sources, including the UCSC genome database [23], Chado databases [24], Ensembl DAS [25], and GenBank XML [26]. In our recent experience, however, the most common sources of genomic information are the laboratories of individual researchers themselves and therefore we focused our attention on direct loading of genomic data files. The system accepts results of computational genomic analyses in the standard, widely used file formats GFF3 (Generic File Format, a *de facto *standard for sharing analysis results), SAM (Sequence Alignment/Map, accepted standard for efficient representation of high throughput sequencing alignments [27]), BAM (binary version of SAM), and BigWig (a binary index of 'wiggle' formatted files for the storage of dense, continuous data [28]). The initial server for an organism is typically primed with data using the combined output from a full genome analysis pipeline, such as MAKER [29]. Working with the MAKER developers, a feature that dynamically instantiates a Web Apollo server as the final step in a MAKER run has been implemented. In addition, users may augment pipeline results with other data, either during the initial installation and configuration process (in which case it is stored on the server), or loading them dynamically from a local file or URL during a session. The URL alternative makes it possible for a group of users to share their data without having to add it to the central server, for example to share and display the output from a Galaxy process [30].

*Locating the region of interest*: Due to the highly fragmented nature of low-coverage genome assemblies with hundreds or thousands of scaffolds, selecting a chromosomal region of interest is not always a straightforward task. To assist in locating a region of interest users may deploy the 'Search Sequence' tool, which queries the assembled genome with a gene or chromosomal region of interest using a BLAT search (BLAST-like Alignment Tool [31]). This feature was implemented using a plug-in architecture, allowing support for search tools other than BLAT with minor additions to the source code. BLAT may point to multiple potential regions containing the query sequence when paralogs are present, and/or when the gene of interest is split across two or more genomic fragments. This search results in list of regions that a user can then chose from by simply clicking on a region's row to display that region in the browser.

**Figure 2:**
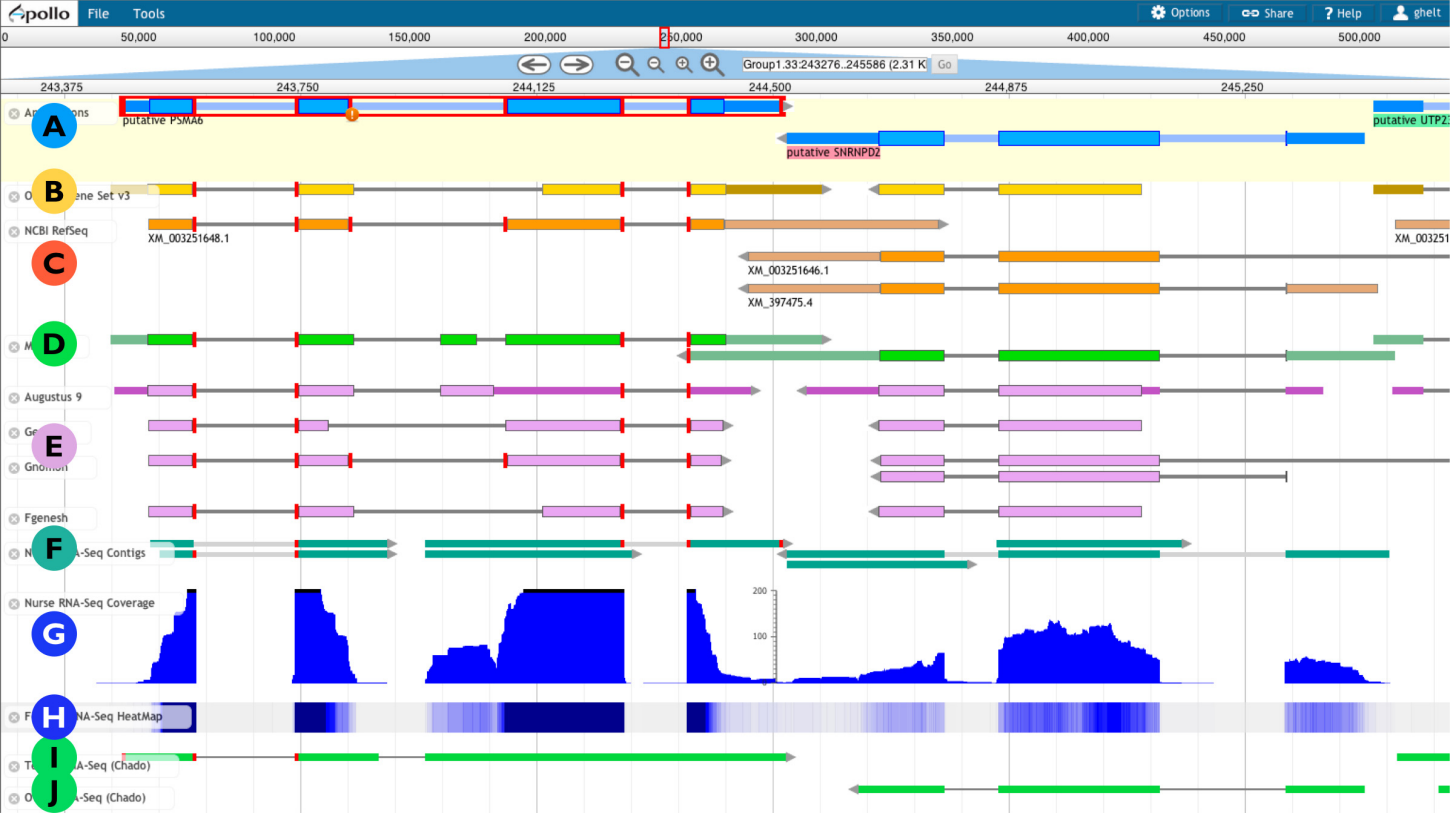
**Example of the Web Apollo interface**. Moving from top to bottom these example tracks from the honeybee (*Apis mellifera*) genome display: **(A) **In-progress gene models interactively being edited by the user. **(B) **The honeybee consortium's official gene set. **(C) **Transcripts from the NCBI RefSeq database. **(D) **Output from MAKER. **(E) **Output from various different gene prediction programs. **(F, I, J) **Contigs generated from RNA-seq data for respectively: nurse bees, testes, and ovaries. **(G) **Coverage map from the nurse bee RNA-seq data. **(H) **RNA-Seq data from forager bees displayed as a 'heat map'. Note that none of the gene predictions are in agreement regarding intron-exon boundaries in (E), which illustrates why manual review is needed. Web Apollo gives biologists the ability to manually resolve disagreements and create a more accurate set of gene predictions to improve upstream analysis pipelines in subsequent runs, as well as provide a more reliable substrate for downstream analyses.

As an example, Figure 2 displays a small region of a scaffold from the honeybee (*Apis mellifera*) genome assembly. Each horizontal track presents a particular type of data, variously shown as graphs, 'heat maps', or as discrete features depending on the type of data and on user preferences. The data tracks retrieved from the server or uploaded by the user are read-only and are used as the evidence to support or refute individual gene models.

*Creating a gene model: *Curators begin the manual annotation process by selecting and dragging the most appropriate computational results into the 'User-created Annotations' area, a writable 'white board' track where they can modify transcripts and individual exons. Alternately there is also the option to automatically promote one of the computational prediction sets. Due to the redundancy of available evidence for highly expressed transcripts, and the fluid growth of the available evidence, we expressly decided not to include any meta-data listing the evidence tracks used to create an annotation. The former would cause the meta-data captured to balloon, and the latter would make it extremely difficult to maintain data integrity. In our experience it is more effective to keep track of dates. If the annotation itself is dated (both for creation and for modification) as well as the evidence, then it is a straightforward operation to compare these and flag discrepancies. It is also important to use the available screen area optimally, particularly as the volume of information increases. Towards this end we added the capacity to restrict the view to a single strand, and to lock the editable white-board track into position so it is visible regardless of whether the user scrolls vertically.

*Modifying a gene model*: Basic editing operations such as deleting, merging, splitting, or duplicating a transcript or part of one, can be accessed from a pop-up menu available for each feature using a right-click of the mouse. To modify exon boundaries, users click to select the subject exon and drag either one of the edges. Apollo facilitates correct determination of exon boundaries by highlighting matching edges across the annotation and evidence tracks, by coloring the CDS annotation and evidence features according to their reading frame (that is, the frame of each exon is indicated by its color, and thus any features with conflicting frames displays in a different color), and by flagging non-canonical splice-sites in the user's annotations. The resulting protein sequence can be used to determine the biological credibility of a gene model by querying highly curated protein databases. Editing requests from different users arrive at the server one at a time (because of the network) and are handled in their order of arrival. The unit of operation includes all the additional edits that are intrinsic to the original operation, that is, if an exon is deleted or shortened then the parent transcript and parent gene are modified as well. The second edit request will either overwrite the first edit, which the first user will be able to see immediately, or in very rare cases of a contradictory edit (for example, an exon being deleted by the first user and then a request to change its boundary by the second user) the second user will receive and error warning, and the annotation will remain as edited by the first user. All operations performed in the 'User-created Annotations' track are recorded in the history and can be reversed or repeated with the 'Undo' and 'Redo' options.

*Exporting data*: To conduct further analyses, users may export their annotations as FASTA-formatted sequences, GFF3 files, or record them in a Chado database.

### Sequence alterations

**Figure 3:**
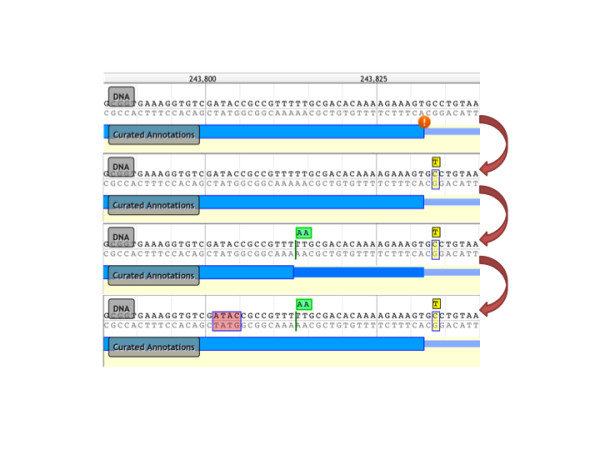
**Example of sequence alteration editing operations**. The top panel shows a transcript annotation (in blue) flagged with an orange exclamation icon indicating that the curated intron-exon junction does not follow a canonical splice site pattern, that is, having a 'GT' immediately 3' of the junction. In the second panel a curator has examined this issue and determined that a base was mis-called in the assembly, and has therefore added a substitution annotation (shown in yellow), substituting a 'T' for a 'C'. This change immediately triggers removal of the non-canonical warning icon, because with the substitution the splice junction now has the canonical 'GT'. In the third panel a curator has created a sequence insertion annotation (shown in green) upstream of the splice, and this leads to a stop codon that truncates the CDS. In the last panel a sequence deletion annotation has been created (shown in red), which causes a frame shift for the annotation transcript, and results in the reversal of the CDS truncation.

During the development of Web Apollo, we encountered a scenario among the newer genome projects that was radically different from our previous experience with large sequencing centers and MODs. The centers and MODs historically focused on assembling reference genomes with deep coverage from Sanger sequencing resulting in full chromosomal assemblies. In contrast, more recent projects are often assembled from Next Generation Sequencing (NGS) technologies which generate shorter reads with higher error rates, resulting in assemblies that are not only more fragmented but also contain a relatively higher number of errors in the genomic sequence [32]. For example, some errors introduced indels in coding sequence, disrupting the reading frame. Biologists needed to annotate the features on the genome, but in order to create the correct transcript annotation, correcting these suspected sequencing and assembly errors was also necessary, and it became a highly requested feature. Curators may now correct suspected assembly errors using Apollo's ability to perform genomic sequence insertions, deletions, and substitutions (Figure 3). These sequence changes do not alter the underlying reference assembly stored on the server, but are maintained as annotations so they can potentially be incorporated into subsequent assemblies for incremental improvements. Within the context of Apollo, these genomic sequence annotations create an underlying virtual sequence that is incorporated when calculating mRNA and protein sequences for these annotations. The resulting sequences can be exported as described below in the Methods section.

### Visualizing stage and cell-type specific transcription

**Figure 4:**
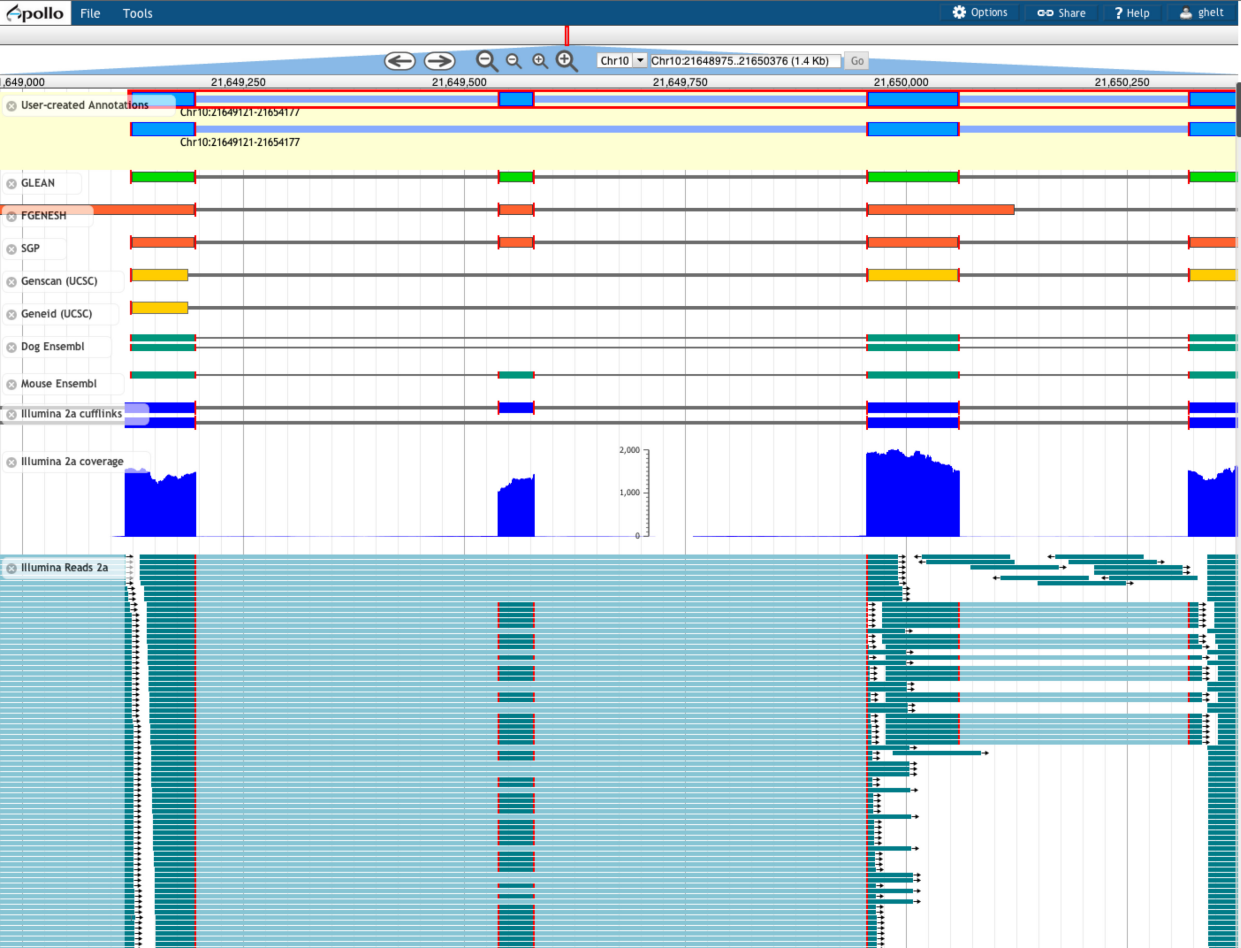
**RNA-Seq evidence provides support for alternative isoforms**. In this example from the bovine genome (*Bos taurus*) the RNA-Seq data was stored as a BAM file and dynamically uploaded. Individual aligned reads are shown in teal. The example highlights the importance of utilizing deep RNA sequencing for curation. Two different splice variants are visible: one variant is visible in the Dog Ensembl track and a different one is visible in the Mouse Ensembl track. The RNA-Seq data track clearly shows evidence that both variants are present in the bovine. Edge-matching (in red) highlights the concordance in exon boundaries between the different tracks.

Using new sequencing technologies researchers are able to capture snapshots of the entire RNA content of samples from particular cell types, particular tissues, at particular developmental stages, or under any number of other specific environmental conditions. These techniques measure expression levels more precisely and offer better opportunities to identify alternate transcripts than the previous methods [33], providing essential information for thorough gene structure annotation. To gain an understanding of expression levels Web Apollo offers multiple modes for transcriptome data visualization, as coverage plots, as 'heat maps', and as alignments. Graphs of expression levels across the genome may be driven from data loaded in BigWig format; alternatively the number of reads per base can be calculated using either the raw sequence data (FASTQ, SFF, and so on) or using alignment data from BAM files. Expression data may also be shown as 'heat map' plots (Figure 2, track H) in which regions with scores above a given threshold acquire a progressively brighter shade of blue, and scores below that threshold progressively become more intensely red. The display of aligned reads (BAM) includes base-by-base alignments for each read, if the MD or CIGAR fields for the read are provided. As shown in Figure 4, Web Apollo can display high-throughput RNA sequencing data from files in any of these formats, either from the server or from user-uploaded data files through a web browser.

### Real-time collaboration

**Figure 5:**
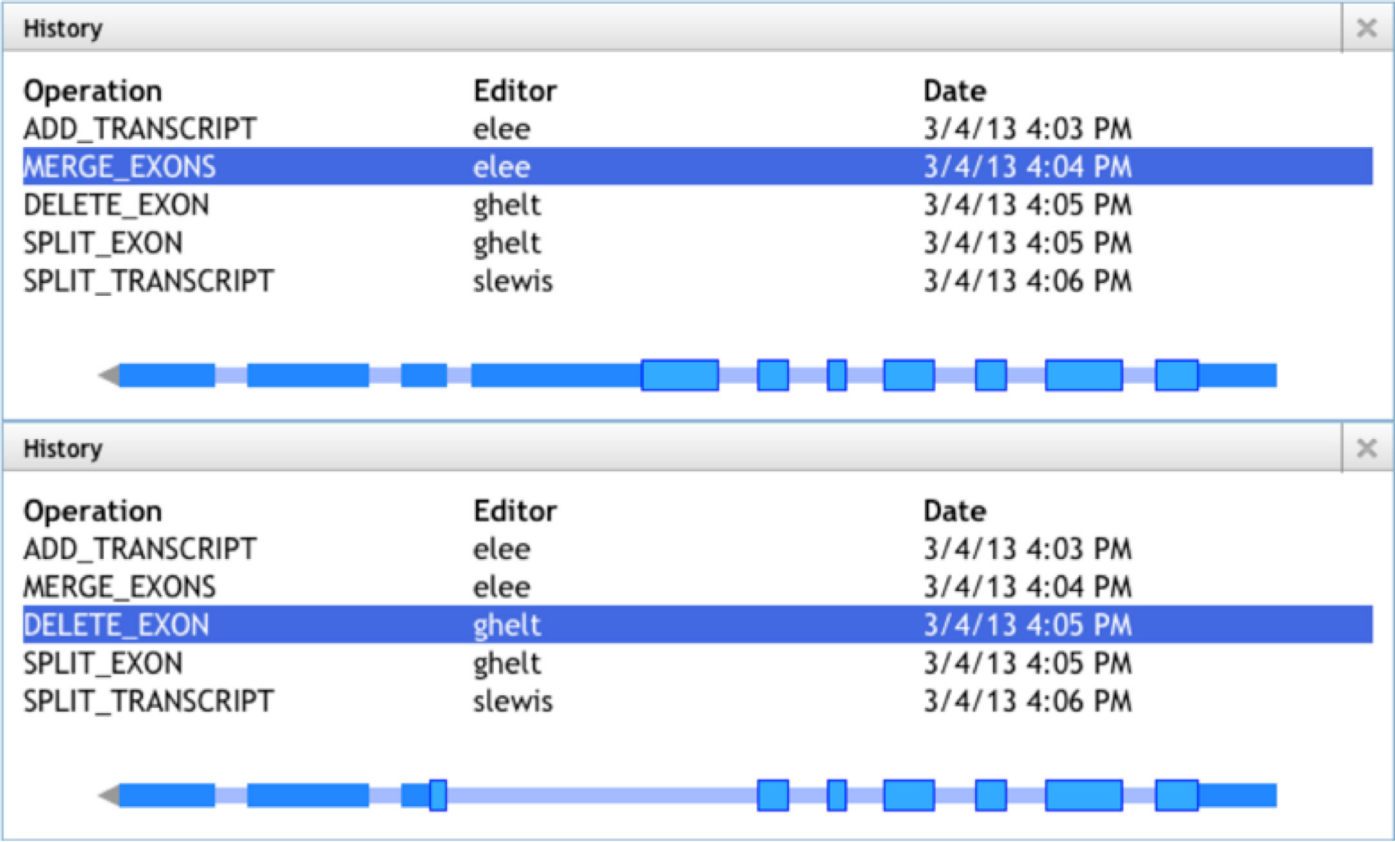
**History tracking and edit operation**. Two History windows show how the transcript changed between edit operations. Each History entry shows the edit operation, the user who made the edit, and the date. The top window shows the transcript after merging of two exons and the one below shows the transcript after an exon has been deleted. Users can click on different History entries, which will display how the transcript looked at that point in time.

In addition to supporting an individual's work, Web Apollo allows groups of researchers to share their annotations and to collaboratively add, delete, and revise annotations. Collaboration is enabled through the server's management of user login, authentication, and editing authorization permissions. The application is flexible enough to support members of a group working concurrently or at different times. Multiple users may work simultaneously on the same region while discussing their work in chat windows or using Voice-over IP services (for example, Skype, Google Hangout, Vidyo, and so on). All changes made in one client are instantly pushed and visible to all other clients. Alternatively, users may work asynchronously, monitoring the changes that occur in their absence. This is possible because the mechanism that supports 'Undo' and 'Redo' functions also supports graphical browsing of an annotation's edit history (Figure 5). Each revision is tracked, dated, and signed so collaborators can visually review the changes and identify the user(s) who made them. Users may add as many details as necessary in support of each annotation in the form of comments. Comments can be chosen from a predefined set, be added as free-text, and/or as cross-references to related resources (for example, gene ontology (GO) functional terms).

*Community adoption: *In the three months since its public release in December of 2012, 18 servers (Table 1) for eight different annotation groups have been set up, some with our group's assistance and others independently.

**Table 1:** List of currently known servers.

Organism	Common name	Group	Annotation status
Acanthamoeba castellanii	Amoeba	BCM-HGSC^a^	Server set up in progress
Helicoverpa armigera	Cotton bollworm	CSIRO^b^	Server set up and analysis in progress
Pythium ultimum	Pythium blight	GMOD	Used one contig to teach a GMOD course
Zea maize	Corn	Iowa^c^	Server set up in progress
Manduca sexta	Goliath moth	Kansas^d^	Server set up in progress
Mayetiola destructor	Hessian fly	Kansas	Server set up and analysis in progress
Acromyrmex echinatior	Leaf cutting ant	Missouri	
Apis mellifera	Honey bee	LBNL/Missouri	Server is available for ongoing annotation
Atta cephalotes	Leaf cutting ant	Missouri	
Bombus impatiens	Eastern bumble bee	Missouri	Used internally to test deployment
Bos taurus	Cow	Missouri	Used internally; new assembly in progress
Cardiocondyla obscurior	Ant, tramp species	Missouri	Community is currently annotating
Monodelphis domestica	Gray, short-tailed opossum	Missouri	Server is available for ongoing annotation
Pogonomyrmex barbatus	Red harvester ant	Missouri	Community is currently annotating
Varroa destructor	Varroa mite	Missouri	Computational gene prediction has begun
Wasmannia auropunctata	Electric ant	Missouri	Community is currently annotating
Pungitius pungitius	Nine spine stickleback	Utah^e^	Analysis in progress
Over 40 species	Pigeons	Utah	Analysis in progress

The list is organized by hosting group. Light gray shading indicates that the group participated in beta testing of this application. Other servers may also be active but we do not track server instances.

^a^Baylor College of Medicine Human Genome Sequencing Center, USA.

^b^Commonwealth Scientific and Industrial Research Organization, Australia.

^c^Iowa State University and United States Department of Agriculture Agricultural Research Service.

^d^Kansas State University Arthropod Genomics Center.

^e^University of Utah Eccles Institute of Human Genetics.

## Discussion

Given that manual annotation is critical to achieving accurate and reliable gene models the issue now becomes how can this process be scaled up to meet the needs of the growing number of genome research projects taking place at smaller facilities and in individual labs. With the shift in sequence data generation, the burden of curation is falling largely on research consortia or *ad hoc *community efforts. Some sequencing centers have supported consortium annotation efforts, either by providing websites for community members to submit annotations (for example, [34-38]), by collaborating with a centralized, external genome annotation group (for example, [39-42]), or by providing Otterlace (for example, [43]). However, more and more often research communities are organizing manual curation efforts among themselves, independent of sequencing centers.

Desktop Apollo gained popularity among smaller groups and over time it became one of the standards used by smaller-scale genome projects in research communities dispersed throughout the world. However, its original design legacy did not make it a perfect fit for the needs of these smaller genome projects. Installation was at times an insurmountable technical hurdle for groups lacking an on-site bioinformaticist. Furthermore, there was no support for automatically sharing annotations among members of the research team. Groups were constrained to saving files to disk and e-mailing these to one another, which is slow, inconvenient, and creates additional bookkeeping work as conflicts were resolved by database curators taking the time to contact the disagreeing annotators individually. With the need to provide a seamlessly integrated annotation flow for smaller teams of researchers in mind we built Web Apollo focusing on support for collaborative annotation efforts. By being browser-based it allows users to see changes made by collaborators working on the same region, in real time, which enables community annotators to quickly resolve issues among themselves directly. Early in the project we made the decision to build the Web Apollo client using the visualization techniques of an existing web-based genome browser, JBrowse [22], the best of the genome browsers alternatives available, thereby lowering overall development costs.

Web Apollo also addresses two key requirements that are particular to the smaller community annotation projects [44,45]. First, recent research communities tend to organize into teams based on areas of biological expertise, often preferring to annotate specific genes or gene families, rather than entire scaffolds. Web Apollo allows users to quickly identify their specific loci of interest by integrating BLAT sequence comparison as an optional entry point. Second, the norm for smaller sequencing efforts is fragmented rather than polished assemblies. Web Apollo scaffold list sorting features provide easy access to scaffolds based on identifiers, even when the assembly consists of tens of thousands of scaffolds.

The establishment of best practices and quality control becomes increasingly important with the wide range of genomic expertise available within different research communities. Research projects must develop appropriate standards given their data and offer some training to assure the success of any community annotation project. The built-in quality control features of Web Apollo are similar to those used in desktop Apollo and other annotation editors such as Otterlace. These include flagging non-consensus splice sites and validating translation of coding sequences. In addition we have developed tutorials and a demonstration site to train users in the gestures required for annotation. Accessibility over the web makes it easy to hold long-distant training sessions.

But perhaps most importantly for the continued improvement of the annotations is that Web Apollo allows continued input to gene annotation as long as a server is maintained for the genome, thus researchers can continue to improve annotations as more data is collected over time. If a research community chooses to follow the 'gatekeeper' approach to community annotation [46], Web Apollo also makes it easy for the gatekeeper to view and revise annotations.

### Future enhancements

As sequencing technologies advance and analytical packages improve, the software providing the visualization and the annotation tools needed for iterative refinement, will necessarily have to keep step. There are a number of natural and powerful extensions to a tool like Web Apollo that will enable more analysis functions to be carried out within a browser.

In the immediate future enhancing the convenience and curatorial utilities for biologists is of central importance. We propose to add the capability to annotate further genomic feature types including cis-regulatory regions, transcription factor binding sites, and non-coding RNAs, along with providing an intuitive way to browse, navigate and visualize these. Another improvement is extending the current methods of accessing data to include data from UCSC [23] and Ensembl [5] by adding support for UCSC data hubs and the Ensembl REST API via the basic JBrowse platform. In addition, the introduction of composite tracks that can utilize multiple data files by integrating metadata about how the files are related, for example sequencing read alignment data in a BAM file and coverage plots derived from those alignments in a BigWig file. This will enable a single track to show sequence read alignments at high zoom levels and transition to showing derived coverage plots at lower resolutions, without the high overhead of dynamically calculating the coverage plot from the alignments. The ability to compose integrated tracks of closely related data, independent of particular input formats, will be extremely useful in other situations, such as a single track combining variant data with background population frequency data. Biologists will also be empowered by enriching feature meta-data to include other attributes, such as description, and status flags in the user's dialog box for editing textual and related identifier information. For example a status flag could be used to signal that a team member requests a review of their annotation. The choice of attributes a curator can edit would be configurable so that each project can decide precisely what meta-data attributes are appropriate for their needs. Other enhancements would offer increased assistance to dispersed research teams, by supporting fine-grained, track-by-track sharing options controlled by the user on the client-side, rather than sharing access coarsely genome-by-genome. This way a researcher can choose with whom to share their individual data tracks (this is available now, but limited to the server side). Most importantly there are several seemingly disparate problems that can be addressed with the same technical solution; challenges such as the fragmented nature of some assemblies, the length of the intronic regions for some genes, and the desire to annotate a single gene family or set of duplicated genes simultaneously. Each of these require that distant regions of the genome be brought into the same visual field - which can be done by synthetically splicing the different regions into a single virtual genome sequence as was done in the Integrated Genome Browser [47], and which our current team of developers have the expertise to implement. As an open-source project we welcome contributions from the community to address these and other natural enhancements to provide a feature-rich, powerful genomic research environment.

Our two over-arching aims are actually two perspectives on the same work. Integration with related community annotation projects whose aims are complementary will enrich the feature set available to the user. Specific integration examples include: (1) establishing interactive, dynamic re-analysis of a particular genomic region using Galaxy or SeqWare [48] for example, rerunning with different analysis parameters; (2) placing a newly predicted protein into a protein family using PANTHER services [49]; (3) using protein family information to examine possible roles a protein may have in particular pathways through interactions with the Reactome pathway annotator [50]; and (4) offering connections to resources such as WikiGenes [51] or RFAM:Wikipedia [20] which focus on capturing more textual types of information.

From a targeted audience point of view actively working with researchers in a wide variety of domains will ensure that Apollo is responsive to biologist's requirements and meets their needs. For smaller genome research investigations ease of installation, an enriched set of annotation capabilities and integration with other community annotation projects are key. We also envision Apollo's increased use in educational and classroom settings. This is one motivation for emphasizing integration with analytical pipeline services such as Galaxy and providing tutorials, training, and annotation guidelines. Lastly, Apollo can support research groups whose focus is exploring genotype to phenotype correlations for the study of human disease. For this group we have already implemented some initial prototypes for enhanced visualization of sequence polymorphisms and variation data, and mockups for allelic frequency and dynamic visualization of the effect or impact a set of variants may have on functional genomic elements. For each of these domains we will continue to take a user-centered design approach and directly engage with the researchers in these areas through future iterations of the framework, as well as with software developers who can contribute to the overall platform.

The current challenge is scaling to accommodate the growing amount of work. These projects must operate using a new paradigm, requiring new software workflows and training in the nuances of genomic annotation. A framework that can enable any individual researcher to generate their own sequence data, run an analysis pipeline using a remote service to analyze their organism of interest, and ultimately generate their own models to publish. Web Apollo represents a major step toward achieving the goal of an integrated genomic analysis environment. It provides a comprehensive toolbox to biologists for manually annotating the features of the genome(s) they are investigating.

## Methods

Web Apollo is comprised of three components: a web-based client, an annotation editing server, and a server-side data service that provides the client with data from different files and databases (Figure 1). These three software components are open source and available free of charge.

### Web-based client

Web Apollo uses JBrowse as its visualization component. JBrowse is a JavaScript-based genome browser that provides a fast, highly interactive interface for the visualization of genomic data on the Web. It handles most rendering of data within the web browser using a combination of standard HTML 'div' and 'canvas' elements, in contrast to traditional web-based genome browsers where the server renders the data as an image and sends that image to the client for display. It also heavily utilizes asynchronous and partial (lazy) data loading. These strategies allow for very dynamic zooming and scrolling. In addition to visualizing many types of data on the server, it allows direct uploading of data from local or remote BAM, GFF3, and BigWig files via the user's web browser. Because JBrowse and Web Apollo are active projects whose development is coordinated, the decision to base Web Apollo visualization on JBrowse has the significant added benefit of leveraging ongoing improvements in JBrowse. For example, during the course of Web Apollo development, the JBrowse team added support for powerful metadata-based annotation track searching, which Web Apollo was able to seamlessly incorporate. Conversely, developments in Web Apollo have both significantly influenced, and in some cases been directly incorporated into JBrowse. For instance, direct display of BAM data was initially implemented in Web Apollo, then revised, improved, and incorporated into JBrowse. Also, the initial design of the JBrowse plug-in system was driven by the needs of Web Apollo. As such, JBrowse plug-ins can extend and alter nearly every aspect of JBrowse's behavior, such as adding new track types, inserting menu items, adding Cascading Style Sheet (CSS) rules for customizing the display, using plug-in-specific images for graphics, and even interacting with other plug-ins that may be available. As a plug-in, the Web Apollo client augments the standard JBrowse feature tracks to support multiple feature selection (including any combination of features and subfeatures), selection highlighting, and edge matching, which can highlight the left or right edge of any feature that match the start or end genome coordinate of a selected feature. Two entirely new track types are also implemented, a gene annotation track ('User-created Annotations' track, A in Figure 2.) and a sequence alteration track ('DNA' track in Figure 3). The 'User-created Annotations' track provides users with the ability to manipulate elements and edit annotations; these manipulations include dragging and dropping features from other tracks to create or modify transcripts, dragging exon edges to change exon boundaries of existing annotations, and using context-specific menus to modify annotations. The 'DNA' track provides user with the ability to create and edit sequence alterations, and also implements rendering of DNA residues and six-frame protein translation. Both tracks connect asynchronously to the annotation-editing server to retrieve existing annotations, send edit requests, and receive edit notifications (Figure 1, arrows).

### Annotation-Editing Engine

The Annotation-Editing Engine is written in Java. It handles all the necessary logic for editing and deals with the complexities of modifications in a biological context, where a single change can have multiple cascading effects (for example, splitting or merging transcripts). The Annotation-Editing Engine currently supports: (1) adding and deleting transcripts; (2) merging and splitting transcripts; (3) manually setting the translation start for a transcript (otherwise the longest ORF is automatically calculated with every edit); (4) flipping the strand for a transcript; (5) adding and deleting exons from existing transcripts; (6) changing exon boundaries; and (7) merging and splitting exons, including the ability to search for canonical splice sites to create a biologically relevant intron when splitting an exon. The Annotation-Editing Engine uses a plug-in architecture, which assists in the identification of isoforms wherever overlapping transcripts are present; the architecture allows groups to configure customized rules to determine whether two transcripts should come from the same gene or from separate ones. Currently, we provide options for 'no overlap' (every transcript comes from a separate gene regardless of whether it overlaps another transcript), 'simple overlap' (a transcript is considered an isoform if it has any overlap with an existing transcript), and 'ORF overlap' (a transcript is considered an isoform only if it overlaps another transcript's coding region, in the same frame). Lastly, as previously described in the 'Sequence alterations' section of the Results, the Annotation-Editing Engine also supports editing of genomic insertions, deletions, and substitutions.

Edits are stored persistently in the server, allowing users to quickly recover their data in the event of unexpected browser or server crashes. We employ a two-stage editing approach. First, data are stored in a BerkeleyDB database for live edits, which provides very responsive storage and retrieval of annotations. Edit histories are also stored in the BerkeleyDB database. Later, after they have been reviewed, these edits can be exported to different formats for further analysis or for non-Web Apollo specific storage. The data exporters also implement a plug-in based architecture that allows easy addition of new exporters. Currently, we support exporting annotations to FASTA, GFF3, and Chado.

In a multiple user environment, user permissions and authentication are important. The server offers multiple levels of user permissions, allowing project owners to decide with whom to share their work, and whether to allow read-only or read-and-write access. User authentication implements a plug-in based architecture, allowing users to adopt their own authentication back-end if needed. We currently support authentication through either a Web Apollo specific SQL database or through Mozilla's Persona authentication service [52]. The server supports multiple, concurrent users through synchronized updates over multiple browser instances, so that every edit is immediately visible to all users who are viewing or editing the same region. The server employs the Comet model to allow the server to push data to clients in real time. The client and server use a long held HTTP connection and when edits are made, the server pushes these updates to the client without it having to explicitly request them.

The server also allows searching of genomic sequences. Its plug-in based architecture allows any number of searching strategies to be used without having to modify the searching framework. Currently Web Apollo supports BLAT for nucleotide and translating searches.

### Server-side genomic data service

Two different server-side genomic data services provide data to the Web Apollo client; one is static and one dynamic. The first is a modified version of the JBrowse data pipeline, a set of Perl scripts that support conversion of analysis data in GFF3 and BED formats to JavaScript Object Notation (JSON [53]) files compatible with the Web Apollo. This conversion is performed once, and the JSON files are stored and served to the web-based client as needed. This pipeline is considered static in the sense that the JSON files are pre-generated before they are needed, and once the JSON files are created the original data files are no longer used.

The second data service we have implemented is a server-side component called Trellis that supports dynamic queries to genomic data sources over HTTP. Trellis is implemented as a Java servlet and uses plug-in architecture for both data sources and output formats. Data source plug-ins are implemented for directly querying the UCSC MySQL database, the Chado Postgres database, and servers supporting the Distributed Annotation System (DAS) protocol [25]. An output plug-in converts responses to the JBrowse JSON format used by the Web Apollo client. This service is considered dynamic because if the data source is updated with new data, the JSON returned will reflect this.

### Testing

We tested server installation and the user interface using new genome assemblies and computed evidence data for *Apis mellifera *(honey bee) and *Bombus impatiens *(bumble bee), contributed by the Honey Bee and Bumble Bee Genome Sequencing Consortiums. We performed additional testing and created a demonstration instance, available at [54], using published bovine genome data [39]. The test datasets from real consortiums allowed us to develop solutions to several formatting issues that may otherwise be problematic in future installations. The sources of gene prediction evidence included NCBI Gnomon [55], Ensembl [15], GLEAN [56], MAKER [29], N-SCAN [57], Fgenesh, Fgenesh++ [58,59], Augustus [60], Geneid [61], and SGP2 [62]. Protein homolog alignments had been generated by Exonerate [63]. Alignments of Sanger-sequenced ESTs and contigs were generated by Exonerate, GMAP [64] or Splign [65]. Alignments of RNASeq data were from TopHat [66].

### Installation options

The original process for setting up the Web Apollo server requires familiarity with server administration, with database administration, and with the applications used by Web Apollo [67]. To facilitate the installation process and assist researchers in overcoming these requirements, we recently developed two solutions. The first is 'GMOD-in-the-Cloud' [68], a virtual machine for deployment on the cloud, which comes with Web Apollo (among other GMOD tools) already installed. This provides a great solution for researchers who do not have any restrictions on hosting their instances and data elsewhere. In addition, for those who manage sensitive data that may need to be kept away from shared spaces and the cloud, we have provided a virtual machine, which can be deployed locally [69].

## Data Access

The first version of Web Apollo was released in December 2012 [70]. At the time of this publication Web Apollo has been downloaded 179 times, from 104 unique IP addresses. Web Apollo is implemented in JavaScript, Java, and Perl, with all major browsers supported. The source code is freely available and maintained in Google Code [71] (server) and GitHub [72] (client). Detailed information can be found online [73], including a user guide [74] and demonstration site.

## Authors' contributions

EL (server side edit engine and client side interactive interface), GH (trellis and client side interactive interface), and RB (basic JBrowse visualization framework) designed, implemented, and deployed the Web Apollo application with contributions from JR (Chado and GFF3 handling) and CC (upstream analysis pipeline, server setups, usability testing, and documentation). MM-T carried out usability testing and wrote the user manual. SL and IH conceived the Web Apollo strategies. CE served as a liaison to user communities and acquired test datasets. SL, CE, LS, and IH supervised the project. All authors discussed the results and implications and commented on the manuscript. All authors read and approved the final manuscript.
